# ERα Signaling Is Required for TrkB-Mediated Hippocampal Neuroprotection in Female Neonatal Mice after Hypoxic Ischemic Encephalopathy^1^,^2^,^3^

**DOI:** 10.1523/ENEURO.0025-15.2015

**Published:** 2016-01-28

**Authors:** Ulas Cikla, Vishal Chanana, Douglas B. Kintner, Eshwar Udho, Jens Eickhoff, Wendy Sun, Stephanie Marquez, Lucia Covert, Arel Otles, Robert A. Shapiro, Peter Ferrazzano, Raghu Vemuganti, Jon E. Levine, Pelin Cengiz

**Affiliations:** 1 Waisman Center, University of Wisconsin School of Medicine and Public Health, Madison, Wisconsin 53705; 2 Department of Neurological Surgery, University of Wisconsin School of Medicine and Public Health, Madison, Wisconsin 53792; 3 Department of Statistics and Bioinformatics, University of Wisconsin School of Medicine and Public Health, Madison, Wisconsin 53792; 4 Department of Neuroscience, University of Wisconsin School of Medicine and Public Health, Madison, Wisconsin 53705; 5 Department of Pediatrics, University of Wisconsin School of Medicine and Public Health, Madison, Wisconsin 53792

**Keywords:** 7,8-dihydroxyflavone, estrogen receptor alpha, hypoxia-ischemia, neonate, src, tyrosine kinase B

## Abstract

Male neonate brains are more susceptible to the effects of perinatal asphyxia resulting in hypoxia and ischemia (HI)-related brain injury. The relative resistance of female neonatal brains to adverse consequences of HI suggests that there are sex-specific mechanisms that afford females greater neuroprotection and/or facilitates recovery post-HI. We hypothesized that HI preferentially induces estrogen receptor α (ERα) expression in female neonatal hippocampi and that ERα is coupled to Src family kinase (SFK) activation that in turn augments phosphorylation of the TrkB and thereby results in decreased apoptosis. After inducing the Vannucci’s HI model on P9 (C57BL/6J) mice, female and male ERα wild-type (ERα^+/+^) or ERα null mutant (ERα^−/−)^ mice received vehicle control or the selective TrkB agonist 7,8-dihydroxyflavone (7,8-DHF). Hippocampi were collected for analysis of mRNA of ERα and BDNF, protein levels of ERα, p-TrkB, p-src, and cleaved caspase 3 (c-caspase-3) post-HI. Our results demonstrate that: (1) HI differentially induces ERα expression in the hippocampus of the female versus male neonate, (2) src and TrkB phosphorylation post-HI is greater in females than in males after 7,8-DHF therapy, (3) src and TrkB phosphorylation post-HI depend on the presence of ERα, and (4) TrkB agonist therapy decreases the c-caspase-3 only in ERα^+/+^ female mice hippocampus. Together, these observations provide evidence that female-specific induction of ERα expression confers neuroprotection with TrkB agonist therapy via SFK activation and account for improved functional outcomes in female neonates post-HI.

## Significance Statement

Female neonate brains are more resistant to the effects of hypoxia-ischemia (HI). We report a novel mechanism that involves the female-biased induction of ERα expression in the hippocampus post-HI, coupled to activation of a cytoplasmic kinase (src) and increased TrkB phosphorylation in the presence of a TrkB agonist. Thus, the enhanced TrkB receptor signaling resulting from this crosstalk mechanism confers decreased programmed cell death in response to TrkB agonist treatment in female versus male subjects. These results clearly demonstrate a role for ERα in enhancing TrkB activation and may account for the relative resistance of the female neonate brain to HI.

## Introduction

Perinatal asphyxia resulting in hypoxia-ischemia (HI)-related brain injury leads to severe, life-long morbidities in thousands of neonates and children born in the U.S. each year (Ferriero, 2004; Nelson and Lynch, 2004; Drobyshevsky et al., 2007; Hill and Fitch, 2012). The physical, emotional, and economic toll taken by these adverse early childhood events is incalculable. Interestingly, clinical studies indicate that male neonate brains are more susceptible to the effects of perinatal asphyxia (Vannucci and Hurn, 2009; Hill and Fitch, 2012) resulting in greater long-term cognitive deficits compared with females with comparable brain injury (Marlow et al., 2005; Tioseco et al., 2006; Hill and Fitch, 2012). In addition, males show increased risk for brain-based developmental disorders including learning disabilities and cerebral palsy compared with females (Donders and Hoffman, 2002; Rutter et al., 2003). The relative resistance of female neonatal brain to adverse consequences of HI suggests that there are sex-specific mechanisms that afford females greater neuroprotection and/or facilitates recovery post-HI (Rutter et al., 2003; Hill and Fitch, 2012). Understanding these mechanisms will help identify sex-specific therapeutic interventions following perinatal HI and lead to better neurological outcomes.

One possible mechanism that could contribute to the sex-based outcomes is differences seen in the expression of estrogen receptors post-HI. Experimental studies have shown that estrogen receptor α (ERα) plays an essential role in the sexual differentiation of neurons in mammalian brain (Simerly et al., 1997) and increased hippocampal expression of ERα results in improvement of memory and learning in aging females long after estradiol (E_2_) therapy is terminated (Witty et al., 2013). In adult female brain after middle cerebral artery occlusion (MCAO), ERα expression is differentially increased at the ischemia site (Wilson et al., 2011). However, the question of whether ERα plays a role in sex differences seen in neonatal brains post-HI has not been investigated.

The development and survival of the mammalian nervous system is largely dependent on the existence of soluble neurotrophic factors. One example is BDNF whose function is primarily mediated through the high-affinity cell-surface receptor TrkB. In the CNS, TrkB is widely expressed in neurons and contributes to diverse biological processes including neuronal survival and neuronal differentiation (Huang and Reichardt, 2001). Following stroke, BDNF signaling through TrkB has been shown to provide neuroprotection through a variety of mechanisms that include neurogenesis and most notably facilitation of anti-apoptosis signaling (Chen et al., 2013). Administration of the BDNF provides neuroprotection in neonatal rats post-HI (Almli et al., 2000) and in excitotoxic lesions resulting in white matter damage in neonates (Husson et al., 2005). Unfortunately, the poor bioavailability of BDNF limits its potential use in clinical trials as a therapeutic intervention (Thorne and Frey, 2001). Alternative small molecules with intrinsic neurotrophic activities and improved bioavailability have promising translational potentials for neuroprotection after brain injury. One example to these small molecules is the selective TrkB agonist, 7,8-dihydroxyflavone (7,8-DHF; Jang et al., 2010). This selective agonist crosses the blood--brain barrier when administered systemically, and provides neuroprotection in MCAO model of adult mice (Jang et al., 2010). In addition, systemically administered 7,8-DHF exerts a profound hippocampal neuroprotection in female but not male mice after perinatal HI (Uluc et al., 2013). It was proposed that this sex-specific neuroprotection was due to the increases in TrkB phosphorylation in female hippocampi.

Estrogen, through its membrane receptor ERα and shared signaling pathways can modulate the actions of BDNF and TrkB in the hippocampus (Scharfman and Maclusky, 2005). Further evidence of an ERα/TrkB link is provided by the finding that, whereas E_2_ therapy induces phosphorylation of TrkB in the adult mouse hippocampus the response is absent in ERα^−/−^ mice (Spencer-Segal et al., 2012). In addition, membrane associated ERα is coupled to the Src family of intracellular kinases called Src family kinases (SFK) and has been shown to facilitate the phosphorylation and activation of TrkB (Barletta et al., 2004; Arpino et al., 2008; Huang and McNamara, 2010). These studies prompted us to study the interaction of ERα and TrkB in the neonatal mice hippocampus post-HI to identify the mechanisms resulting in relative resistance of female neonatal brain to HI and facilitating recovery.

We report a novel mechanism for sexually differentiated hippocampal neuroprotection and identify a potential crosslink between ERα and TrkB in neonatal mice hippocampus post-HI. Our studies test the hypothesis that HI in female neonatal hippocampus preferentially induces ERα expression resulting in sexually differentiated phosphorylation of the TrkB and decreasing apoptosis, with SFK functioning as an intermediary step providing crosstalk between ERα and TrkB.

## Materials and Methods

### Materials

Mouse microtubule associated protein 2 (MAP2) antibody, goat serum, and 7,8-DHF were obtained from Sigma-Aldrich. Rabbit polyclonal anti-phospho-src (Y418), rabbit polyclonal anti-phospho-TrkB (Y515) and rabbit anti-cleaved caspase 3 for immunohistochemistry were purchased from Abcam. Rabbit polyclonal phospho-TrkB (Y705) for immunoblotting was obtained from Signalway Antibody. Rabbit polyclonal anti-TrkB for immunoblotting and mouse monoclonal anti-neuronal nuclei (NeuN) for immunohistochemistry were obtained from EMD Millipore. Rabbit monoclonal anti-src for immunoblotting and rabbit polyclonal anti-cleaved caspase 3 (Asp 175) were obtained from Cell Signaling. Rabbit anti-ERα (MC-20) for immunoblotting was obtained from Santa Cruz Biotechnology. Mouse anti-β-actin was obtained from Developmental Studies Hybridoma Bank. Vectashield mounting media with DAPI was purchased from Vector Laboratories. Goat anti-mouse AlexaFluor 488-conjugated IgG, goat anti-rabbit AlexaFluor 546-conjugated IgG, PlatinumTaq Master Mix, Trizol, Purelink, PCR master mix, TaqMan probes for ERα, ERβ, BDNF and β-actin, trypsin-EDTA, HBSS, horse serum, penicillin/streptomycin, neurobasal medium, and B-27 medium were obtained from Life Technologies.

### Animal use

All procedures on animals were carried out in adherence with the NIH *Guide for the Care and Use of Laboratory Animals* using protocols reviewed by the Institutional Animal Care and Use Committee at our institution.

### Genotyping

ERα heterogeneous (ERα^+/−^) C57BL/6J mice were bred and pups were sexed and genotyped within 9 d of birth. Genotypes were determined by PCR of genomic DNA from finger or toe clippings. Clippings were heated at 95°C for 45 min in 50 mm NaOH and neutralized with equal volume of 1 m Tris, pH 6.8. One microliter of this DNA solution was added to 19 μL of the following: 0.25 μM of primers for the ERα gene, 1× GoTaq Buffer (Promega), 0.2 mm each deoxynucleotide (Promega) and 8 U Platinum Taq (Life Technologies). PCR was performed for 30 cycles as follows: 95°C for 3 min, denaturation at 95°C for 30 s, annealing at 58°C for 30 s (ERα^−/−^ PCR1) or 51°C for 30 s (ERα^−/−^ PCR2), and elongation at 72°C for 1 min. PCR products were separated electrophoretically on an ethidium bromide-containing 2% agarose gel and visualized under UV illumination.

### Induction of neonatal HI

HI was induced as previously described with some modification (Vannucci and Vannucci, 1997). Postnatal day (P) 9 C57BL/6J mice were anesthetized with isofluorane (Butler Schein Animal Health Supply; 3% for induction, 1.5% for maintenance) in 2:1 nitrous oxide-oxygen. The body temperature of the pups were maintained at 36^o^C using a heated surgical table (Molecular Imaging Products). Under a surgical microscope (Nikon SMZ-800 Zoom Stereo, Nikon), a midline skin incision was made and the muscle overlying the trachea visualized. The left common carotid artery was freed from the carotid sheath by blunt dissection, electrically cauterized, and cut. The incision was injected with 0.5% bupivacaine and closed with a single 6.0 silk suture. Animals were returned to their dams and monitored continuously for a 2 h recovery period. To induce unilateral ischemic injury, the animals were placed in a hypoxia chamber (BioSpherix) equilibrated with 10% O_2_ and 90% N_2_ at 36°C for 50 min. After HI, animals were returned to their dams and monitored for pain and discomfort every minute for the first 30 min, every 30 min for the next 2 h, and then daily until sacrificed. This is a well-characterized model of neonatal HI and results in reproducible brain injury ipsilateral (IL) to the electrocauterized left common carotid artery(Vannucci and Vannucci, 1997; Cengiz et al., 2011; Uluc et al., 2013). In this model, unilateral severing of common carotid artery alone does not induce ischemic injury due to collateral circulation from the contralateral (CL) side through the circle of Willis. Only subsequent exposure to hypoxia results in hemispheric ischemia as a result of the preferential decrease of blood flow to the ipsilateral (IL) hemisphere secondary to hypocarbia (Mujsce et al., 1990).

Sham-operated mice received anesthesia and exposure of the left common carotid artery without electrocauterization or hypoxia, as described in this model before (Fang et al., 2013).

### Drug administration

*In vivo*: male and female littermates were randomly divided into HI-vehicle control and HI + 7,8-DHF therapy groups. The HI + 7,8-DHF-treated groups received the initial dose of 7,8-DHF (5 mg/kg, i.p.) diluted in 0.1 m PBS at 10 min after HI. Subsequently, mice were given a daily dose for 2 d after HI. The HI-vehicle control groups received an equal volume of PBS at the same time points.

### Brain tissue preparation

On day 3 (P12) or day 1 (P10) post-HI, animals were either decapitated for collection of fresh tissue or perfusion fixed *in situ*. For perfusion fixation, the animals were anesthetized with isoflurane as described and transcardially perfused with 4% paraformaldehyde and decapitated. After postfixation of the brains in 4% paraformaldehyde overnight, brains were stored in a 30% sucrose/PBS solution for 48 h and then sectioned (35μm thick) on a freezing sliding microtome (Leica SM2000R). The brain sections were then cryoprotected in an antifreeze solution for storage at −20°C.

### Double-immunofluorescence staining

After rinsing with 0.1M Tris-buffered saline (TBS), brain slices were incubated with TBS^++^ (0.1% Triton X-100 and 3% goat serum in 0.1 m TBS) for 30 min at 37°C. Slices were double-stained with anti-MAP2 (1:500) and anti- p-TrkB^Y515^ (1:100) or anti-cleaved caspase 3 (1:200) and anti-NeuN (1:200), or anti-p-src^Y418^ (1:250) and anti- NeuN (1:200), for 1 h at 37°C and then overnight at 4°C. After washing with TBS (3 × 10 min), brain sections were incubated for 1 h at 37°C with goat anti-rabbit AlexaFluor 488-conjugated IgG (1:200) and goat anti-mouse AlexaFluor 546-conjugated IgG (1:200) in TBS^++^. Following rinsing with TBS, the slices were mounted on slides using Vectashield mounting media with DAPI. Primary antibodies were eliminated in some slices to control for nonspecific secondary antibody staining. Slides were imaged with either a Nikon A1R-Si confocal microscope using a 20× objective or whole-brain images collected with a Zeiss epifluorescent microscope using a 5× objective and Stereo Investigator software (MBF Bioscience).

### Quantification of pTrkB immunofluorescence staining

To semi-quantitate pTrkB^Y515^ staining, whole-brain images were imported into Image J software (Schneider et al., 2012) and a region-of-interest was drawn around the CL and IL hippocampus. The mean pixel values for the CL and IL region of interests were subtracted from background pixel values and expressed as the IL/CL ratio. Three slices through the hippocampi from each brain were analyzed and the IL/CL ratios averaged.

### Immunoblotting

The hippocampal protein expression of total TrkB, p-TrkB ^Y705^, total src, p-src^Y418^, and ERα at 3 d and cleaved caspase-3 (c-caspase-3) at 1 d post-HI were quantified. The CL and IL hippocampi from two brains were harvested and pooled in order to achieve the adequate protein content. The tissues were homogenized and centrifuged at 2200 × *g* for 5 min at 4°C. The protein content was determined by the bicinchionic acid method (Pierce). The protein samples (50 μg) and pre-stained molecular mass markers in a SDS buffer were electrophoretically separated on 4–20% gradient SDS gels. The resolved proteins were electrophoretically transferred to a nitrocellulose membrane. After incubation in 5% nonfat dry milk in TBS for 1 h, the blots were probed with the anti-TrkB antibody (1:1000) and anti-p-TrkB^Y705^ antibody (1:800); or anti-t-src (1:1000) and anti-p-src^Y418^ (1:800); or anti-ERα (1:500) and β-actin (1:2000); or c-caspase-3 (1:800) and β-actin (1:2000) overnight at 4°C. After rinsing with TBS, the blots were incubated with goat anti-rabbit horseradish peroxidase-conjugated secondary IgG (1:6000) or rabbit anti-mouse horseradish peroxidase-conjugated secondary IgG (1:6000) for 1 h. Bound antibodies were visualized using an enhanced chemiluminescence assay (Millipore or Biorad).

### Quantitative PCR

CL and IL hippocampi were freshly harvested and immediately placed on dry ice. Total RNA was extracted from single hippocampi using Trizol and a Purelink kit from Life Technologies. The amount of total RNA was determined from the optical densities measurements at 260 and 280 nm (Nanodrop, Thermo Scientific). The reverse transcription reaction (RT) was performed using SuperScript III reverse-transcription reagents and protocols from Life Technologies. Two micrograms of total RNA, random hexamers, oligdTs, dNTPS, and RT buffer were heated for 10 min at 65° for denaturation. First Strand Buffer, DTT, RNAase inhibitor, and the superscript reverse III transcriptase were then added and the samples heated up to 42°C for 1 h followed by 5 min at 95°C to inactivate the reverse transcriptase. The resulting cDNA samples were then diluted 1:10 with RNAase free water and stored at −80°C until use. For quantitative PCR (qPCR) amplification ∼20 ng of cDNA was used. Each reaction (final volume 19 μl) for a single gene was done in duplicate and consisted of predesigned gene-specific primers and probes for ERα (Mn00433149_m1), ERβ (Mm00599821_m1), BDNF (Mm04230607_s1), or β-actin (Mm00607969_s1) and TaqMan Master Mix on a 96-well plate. PCR amplification was accomplished using an Applied Biosystem 7500 qPCR system (Life Technologies) running a standard amplification protocol (50°C 2 min, 95°C 10 min, 95°C 15 sec, 60°C 1min, 40 cycles). Relative gene expression was calculated by the 2^−ΔΔ^*^C^*_T_ method (relative to female CL) or a standard curve for the control gene (β-actin) was obtained by serial 1:10 dilution of the cDNA from the female CL hippocampus and the undiluted sample assigned a value of 1. Cycles to threshold values were analyzed using the System 7500 SDS software (Life Technologies). The calculated values were normalized to their corresponding β-actin values and expressed relative to the value for the female CL hippocampus.

### Statistical analysis

ANCOVA was used to evaluate the effect of sex, HI, and 7,8-DHF therapy on all immunoblot band density parameters (p-TrkB^Y705^/f-TrkB, f-TrkB/β-actin, t-TrkB/β-actin, i-TrkB/β-actin, p-src^Y418^/t-src, and c-caspase-3/β-actin). The primary outcome variables were the immunoblot band density parameters from the IL side. The analyses were adjusted by including the corresponding CL side values as covariates in the model. Thus, in these figures we report the adjusted IL mean ± SEM. The main and interaction effects between factors were evaluated. *p* values were calculated from the corresponding *F* values of the main or interaction effects. The comparisons of the outcome parameters between males and females were made by constructing sliced contrast matrices of the corresponding two- or three-way interaction effects. Analogously, ANCOVA was conducted to evaluate the sex effect for HI treated subjects on BDNF and ERα expression levels. The results were presented using bar charts displaying the adjusted means and corresponding standard error bars. Analysis of variance was performed to evaluate the effects of sex and mutation status on the percentage change in the IL/CL ratio of hippocampal pTrkB^Y705^ staining levels. All *p* values are two-sided and *p*<0.05 was used to define statistical significance. Statistical analyses were conducted using SAS software (SAS Institute) version 9.3 (**Table 1**).

**Table 1.** Statistical table

	Data structure	Type of test	95% Confidence interval
a	Normally distributed	ANCOVA, post-test comparison	0.97–1.47
b	Normally distributed	ANCOVA, post-test comparison	0.54–0.93
c	Normally distributed	ANCOVA, post-test comparison	0.15–0.60
d	Normally distributed	ANCOVA, type III test interaction	NA
e	Normally distributed	ANCOVA, type III test interaction	NA
f	Normally distributed	ANCOVA, post-test comparison	0.14–0.24
g	Normally distributed	ANCOVA, post-test comparison	0.16–0.22
h	Normally distributed	ANCOVA, post-test comparison	0.12–1.10
i	Normally distributed	ANCOVA, post-test comparison	0.13–0.61
j	Normally distributed	ANCOVA, post-test comparison	0.38–0.90
k	Normally distributed	ANCOVA, post-test comparison	0.04–0.18
l	Normally distributed	ANCOVA, post-test comparison	0.14–0.32
m	Normally distributed	ANCOVA, post-test comparison	0.27–0.35
n	Normally distributed	ANCOVA, post-test comparison	0.05–0.19
o	Normally distributed	ANCOVA, post-test comparison	0.04–0.20
p	Normally distributed	ANCOVA, post-test comparison	0.65–0.79
q	Normally distributed	ANCOVA, post-test comparison	0.63–1.09
r	Normally distributed	ANCOVA, post-test comparison	−0.03 to 0.33
s	Normally distributed	ANCOVA, post-test comparison	0.22–1.32
t	Normally distributed	ANCOVA, post-test comparison	0.24–0.36
u	Normally distributed	ANCOVA, post-test comparison	0.42–0.53
v	Normally distributed	ANCOVA, post-test comparison	0.15–0.26
w	Normally distributed	ANCOVA, post-test comparison	0.15–0.27

## Results

### HI induces increased ERα mRNA and protein expressions in female but not in male hippocampi

**Figure 1.**
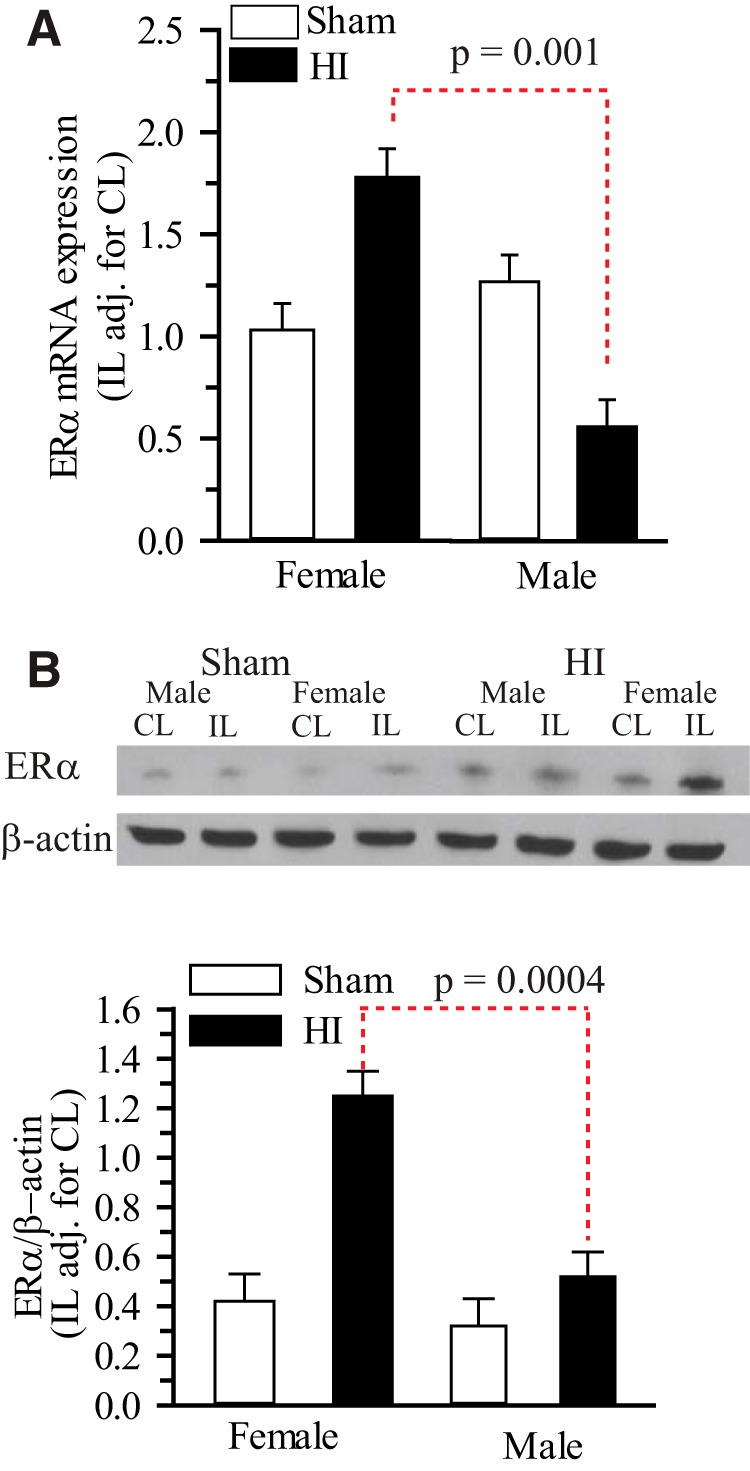
**ERα mRNA and protein expressions in hippocampus at 3 d post-HI.** ERα^+/+^ mice were subjected to either sham operation or HI. Three days, later the male and female hippocampi were probed for the quantification of ERα mRNA and ERα protein expressions. ***A***, Hippocampal ERα mRNA expression was quantified 3 d post-HI using qPCR. Data is IL expression relative to sham-operated female, adjusted for CL ± SEM, *n* = 5–6. *n*=number of pups. ***B***, Top, Representative blot of ERα protein expression in the IL and CL neonatal hippocampi from male and female mice subjected to either sham operation or HI. Blot was probed for β-actin as a loading control. Bottom, Summary figure of the ERα/β-actin ratio in male and female hippocampi of sham and HI mice. Data are mean IL adjusted for CL ± SEM, *n* = 3–4. *n* = number of blots per group (2 pups per group).

ERα mRNA and protein expression were increased in the ischemic cortex of ovariectomized adult female rats, but were unchanged in adult males in an MCAO model (Westberry et al., 2008; Wilson and Westberry, 2009). However, ERα mRNA and protein expression are unknown in the newborn hippocampus following HI. To answer the question of whether ERα plays a role in sex differences seen in neonatal brains post-HI, we quantified ERα mRNA and protein expression in hippocampi of both male and female neonate mice 3 d post-HI. Using qPCR and immunoblotting, we detected 3.1-fold greater ERα mRNA (Fig. 1*A*) and 2.4-fold greater ERα protein (Fig. 1*B*) expression in the IL hippocampus of female mice compared with male mice 3 d post-HI (*p* = 0.001^a^ and *p*=0.0004^b^, respectively) (Table 1). ERβ mRNA expression levels were below the limit of detection (*C*_t_ values > 35) in both CL and IL hippocampi obtained from sham-operated and post-HI mice in both sexes. The sexually differentiated up-regulation of ERα prompted us to further investigate the role of ERα in TrkB phosphorylation both in ERα^+/+^ and ERα^−/−^ mice.

### TrkB agonist therapy enhances HI-induced hippocampal TrkB phosphorylation in neonatal females, but not in males

**Figure 2.**
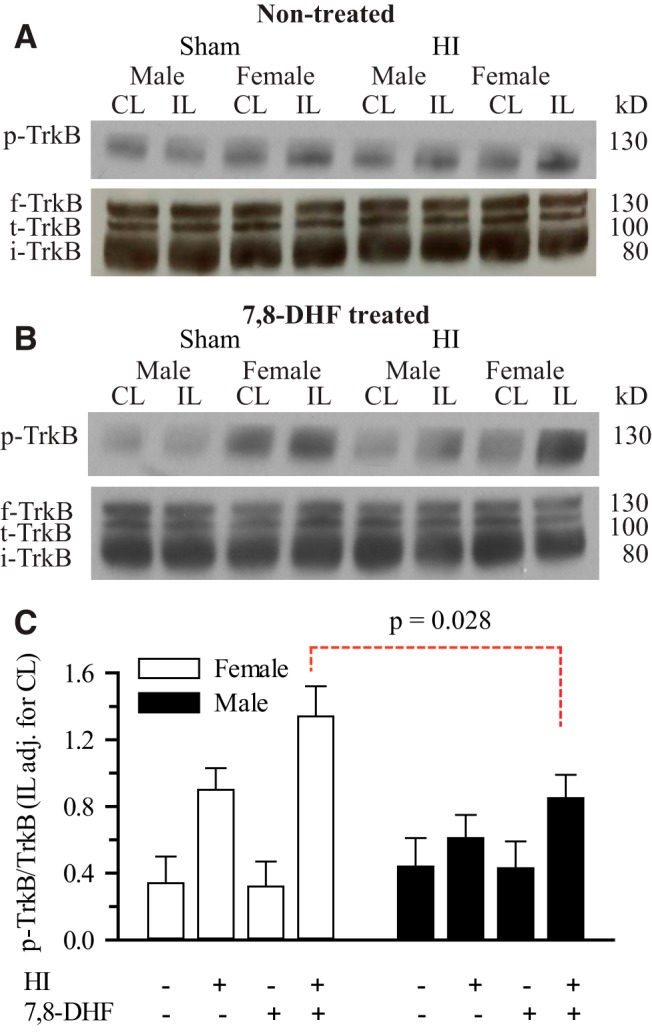
**Effect of HI and 7,8-DHF on hippocampal TrkB phosphorylation in ERα^+/+^ mice.** ERα^+/+^ mice were subjected to either sham operation or HI, with or without 7,8-DHF treatment. Three days later, the male and female hippocampi from the CL and IL sides were probed for phosphorylated TrkB (p-TrkB) expression, full-length TrkB (f-TrkB), truncated TrkB (t-TrkB), immature TrkB (i-TrkB), or degraded TrkB (d-TrkB). ***A***, Representative blots of p-TrkB, and f-TrkB, t-TrkB, i-TrkB in the neonatal hippocampi of sham and HI ERα^+/+^ mice without 7,8-DHF treatment. ***B***, Representative blots of p-TrkB, and f-TrkB, t-TrkB, i-TrkB in the neonatal hippocampi of sham and HI ERα^+/+^ mice with 7,8-DHF treatment. ***C***, Summary figure of the p-TrkB/f-TrkB ratio in female and male hippocampi of sham and HI ERα^+/+^ mice with and without 7,8-DHF treatment. Data are mean IL adjusted for CL ± SEM, *n* = 3–4. *n* = number of blots per group (2 pups per group).

Uluc et al. (2013) recently reported that 7,8-DHF triggers a significant sex-dependent neuroprotection in female neonatal mice hippocampi post-HI. However, the baseline and post-TrkB agonist treatment levels of p-TrkB in sham-operated male and female neonatal mice were not assessed. To investigate the levels of hippocampal p-TrkB with and without 7,8-DHF therapy in sham-operated mice, we sham-operated P9 mice and determined levels of p-TrkB^Y705^ 3 d after sham operation. We could not detect any sex differences in hippocampal TrkB^Y705^ phosphorylation between treatment groups in sham-operated neonatal mice **(** Fig. 2*A*–C**)**. Similar to the results of Uluc et al. (2013), we detected significantly higher hippocampal TrkB phosphorylation in 7,8-DHF-treated females compared with males at 3 d post-HI (1.34 ± 0.18 vs 0.85 ± 0.14, *p*=0.028^c^; Fig. 2*B*,*C*). A significant increase in hippocampal TrkB^Y705^ phosphorylation in mice 3 d post-HI was also detected regardless of the sex and 7,8-DHF therapy (*p*=0.0002^d^). There was also a significant sex and HI interaction (*p*=0.023^e^). This latter response is more robust in females than males and may account for the sexually differentiated neuroprotection observed with TrkB agonist therapy. We then sought to determine whether hippocampal ERα expression is required for TrkB^Y705^ phosphorylation in neonatal mice post-HI or not, using ERα^−/−^ mice.

### HI and TrkB agonist therapy fails to increase the TrkB^Y515^ phosphorylation in ERα^−/−^ mice hippocampus

**Figure 3.**
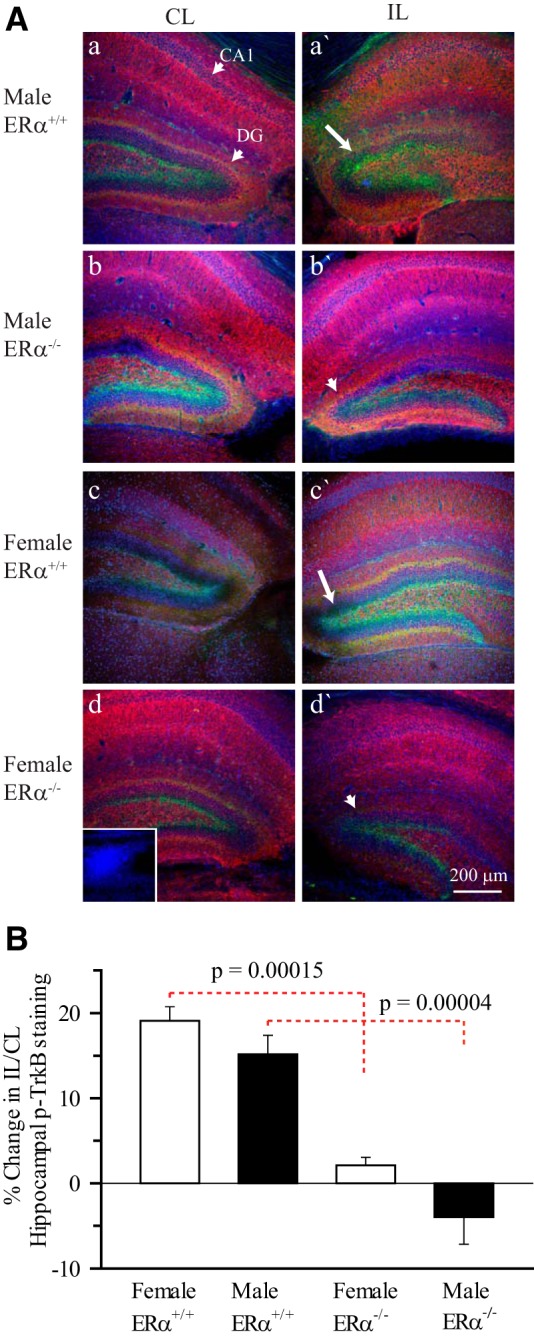
**Immunohistological staining of p-TrkB^Y515^ in ERα^+/+^ and ERα^−/−^ mice 3 d post- HI. *A***, Changes in MAP2 and p-TrkB^Y515^ immunoexpression in the CL and IL hippocampi of ERα^+/+^ and ERα^−/−^ P9 male and female mice were examined 3 d post-HI. MAP2 (red), p-TrkB^Y515^ (green), and DAPI (blue). Arrow, Increased p-TrkB^Y515^ staining. Arrowhead, Decreased p-TrkB^Y515^ in ERα^−/−^ hippocampi. Inset, Primary antibody control. Scale bar, 200 μm. ***B***, Summary figure showing the percent change in IL/CL ratio of hippocampal mean p-TrkB^Y515^ fluorescent intensities in ERα^+/+^ and ERα^−/−^ male and female mice hippocampi 3 d post-HI. IL/CL ratio of p-TrkB^Y515^ obtained from uninjured naïve mouse hippocampus was assumed to be one. Three hippocampal slices per brain were analyzed. Data are mean ± SEM. *n* = 8 for ERα^+/+^ and *n*=3 for ERα^−/−^. *n* = number of pups.

To determine the role of ERα in TrkB phosphorylation, we initially compared the hippocampal p-TrkB^Y515^ expressions in both ERα^+/+^ and ERα^−/−^ mice post-HI by immunostaining. Supporting our *in vivo* immunoblotting studies, semiquantitative analysis of pTrkB immunostaining revealed an ∼17% increase in the IL hippocampal pTrkB^Y515^ immunoreactivity at 3 d post-HI in the hippocampus of both male and female ERα^+/+^ mice (Fig. 3*A*,*B*). The majority of the TrkB phosphorylation detected by immunostaining was in the dentate gyrus (Fig. 3*A*). In contrast, p-TrkB^Y515^ immunostaining was significantly less throughout the hippocampus, including the dentate gyrus in both male (*p* = 0.00004^f^) and female (*p* = 0.00015^g^) ERα^−/−^ mice compared with ERα^+/+^ mice (Fig. 3*A*,*B*). This suggests that ERα is required for the HI-induced increase in hippocampal p-TrkB post-HI.

**Figure 4.**
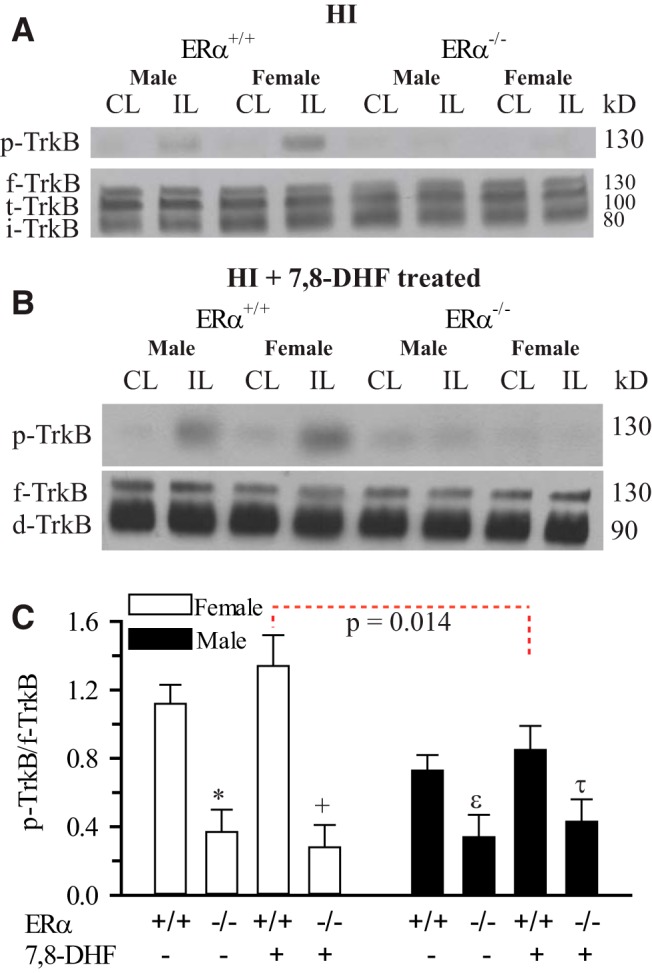
**Effect of HI and 7,8-DHF on hippocampal p-TrkB in ERα^+/+^ and ERα^−/−^ mice.** ERα^+/+^ and ERα^−/−^ mice were subjected to HI with and without 7,8-DHF therapy. Three days later, the male and female hippocampi from the CL and IL sides were probed for p-TrkB^Y705^ expression, f-TrkB, t-TrkB, and i-TrkB. ***A***, Representative blots of hippocampal p-TrkB (top), f-TrkB, t-TrkB and i-TrkB in ERα^+/+^ and ERα^−/−^ mice without 7,8-DHF therapy 3 d post-HI. ***B***, Representative blots of hippocampal p-TrkB (top), f-TrkB, d-TrkB (t-TrkB and i-TrkB) in ERα^+/+^ and ERα^−/−^ mice with 7,8-DHF therapy 3 d post-HI. ***C***, Summary figure of the hippocampal p-TrkB/f-TrkB ratio in ERα^+/+^ and ERα^−/−^ mice with and without 7,8-DHF therapy 3 d post-HI. Data are mean IL adjusted for CL ± SEM, *n* = 3. *n* = number blots per group. *p = 0.018^p^, +*p*= 0.035^q^, ε*p*= 0.334^r^, τ*p* = 0.0488^s^ versus ERα^+/+^.

To confirm our immunostaining findings, immunoblotting experiments were performed to quantify the hippocampal p-TrkB^Y705^/f-TrkB ratio in ERα^−/−^ male and female mice post-HI (Fig. 4). Neither HI nor 7,8-DHF therapy increased the hippocampal TrkB phosphorylation in the ERα^−/−^ mice (Fig. 4). Interestingly, the sexually differentiated hippocampal TrkB phosphorylation observed in female ERα^+/+^ mice was also abolished in ERα^−/−^ mice. Together, these data suggest that there is preferential TrkB phosphorylation in female neonates that depends on the ERα activity.

### Enhanced 7,8-DHF-dependent hippocampal TrkB phosphorylation in females does not depend on expression of TrkB isoforms

**Figure 5.**
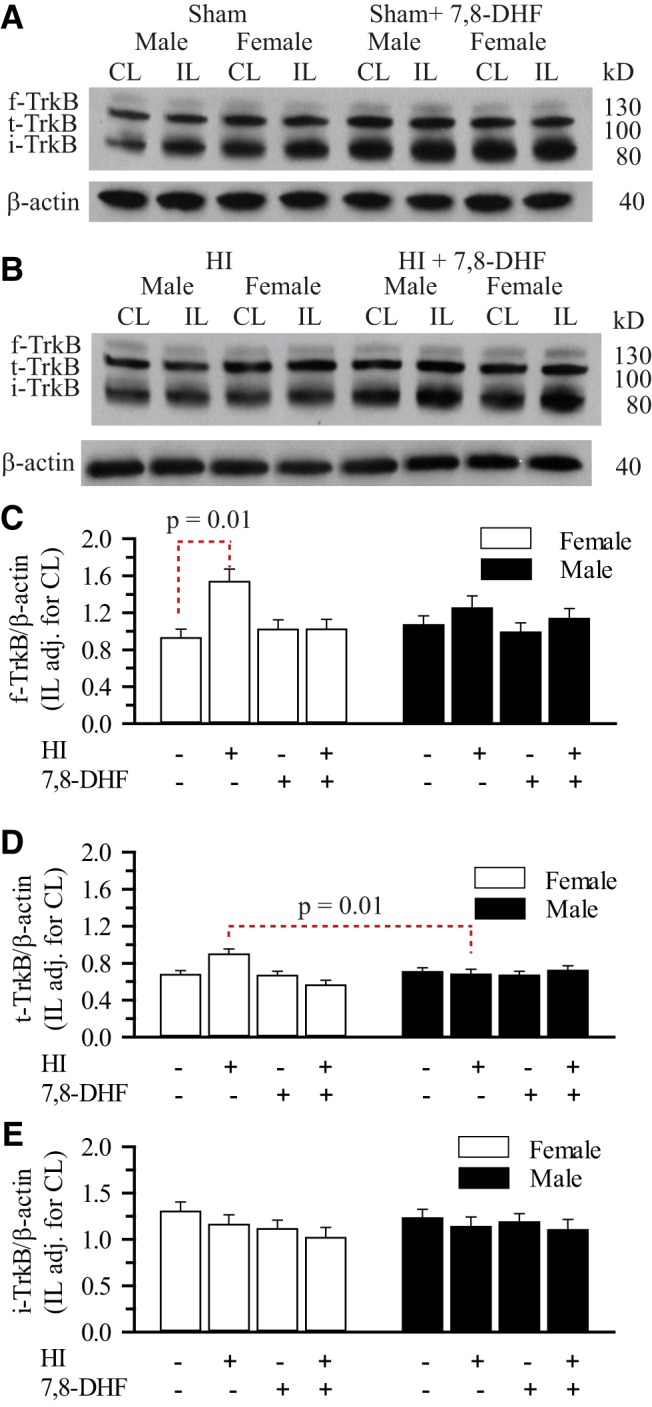
**Effect of HI and 7,8-DHF on hippocampal TrkB subtypes in ERα^+/+^ mice.** The hippocampi of male and female ERα^+/+^ mice were probed for full-length TrkB (f-TrkB), truncated TrkB (t-TrkB), immature TrkB (i-TrkB), and β-actin. ***A***, Representative blots of f-TrkB, t-TrkB, i-TrkB, and β-actin from sham-operated male and female neonatal hippocampi with and without 7,8-DHF therapy. ***B***, Representative blots of f-TrkB, t-TrkB, i-TrkB and β-actin from male and female neonatal hippocampi with and without 7,8-DHF therapy 3 d post-HI. ***C***, Summary figure of the f-TrkB/β-actin ratio in male and female hippocampi with and without 7,8-DHF therapy 3 d post-sham operation or post-HI. Data are mean ± SEM, *n* = 3–4. *n*=number of blots per group. ***D***, Summary figure of the t-TrkB/β-actin ratio in male and female hippocampi with and without 7,8-DHF therapy 3 d post-sham operation or post-HI. Data are mean ± SEM, *n* = 3-4. *n*=number of blots per group. ***E***, Summary figure of the i-TrkB/β-actin ratio in male and female hippocampi with and without 7,8-DHF therapy 3 d post-sham surgery or post-HI. Data are mean IL adjusted for CL ± SEM, *n* = 3–4. *n* = number of blots per group.

TrkB can exist in other isoforms that could affect its signaling capacity. The two dominant TrkB receptor isoforms expressed in the brain are full-length (f-TrkB) and truncated TrkB (t-TrkB). Presence of t-TrkB can prevent f-TrkB phosphorylation in male mouse mammary gland (Liu et al., 2012). In addition, Golder et al. proposed that intermittent hypoxia can result in the synthesis of an immature form of TrkB (i-TrkB) which is hypoglycosylated and when phosphorylated results in ligand-independent TrkB signaling (Golder et al., 2008). Therefore, we determined the levels of f-TrkB, t-TrkB, and i-TrkB in the hippocampi in sham and HI mice with and without 7,8-DHF therapy. As seen in Figure 5, under sham and HI conditions, we detected no difference in f-TrkB and i-TrkB levels between male and female hippocampi with and without 7,8-DHF therapy. There was a trend towards higher f-TrkB levels in female compared with male hippocampi 3 d post-HI without the 7,8-DHF therapy, but did not reach statistical significance (*p*=0.08). There was a statistically significant difference in f-TrkB levels between the female sham and HI animals, this difference did not persist in 7,8-DHF treated animals (*p*=0.01^h^). However, our results did show a statistically higher hippocampal t-TrkB expression in female IL hippocampus (adjusted for CL) post-HI compared with males (*p* = 0.01^i^), this significance did not hold in 7,8-DHF treated females post-HI. More importantly, there was no difference in hippocampal t-TrkB expression between males and females with 7,8-DHF therapy at 3 d post-HI. Thus, the sexually differentiated hippocampal TrkB^Y705^ phosphorylation in response to 7,8-DHF therapy does not appear to be dependent on differential expression of TrkB subtypes post-HI.

### BDNF mRNA expression is decreased in female hippocampus 3 d post-HI

**Figure 6.**
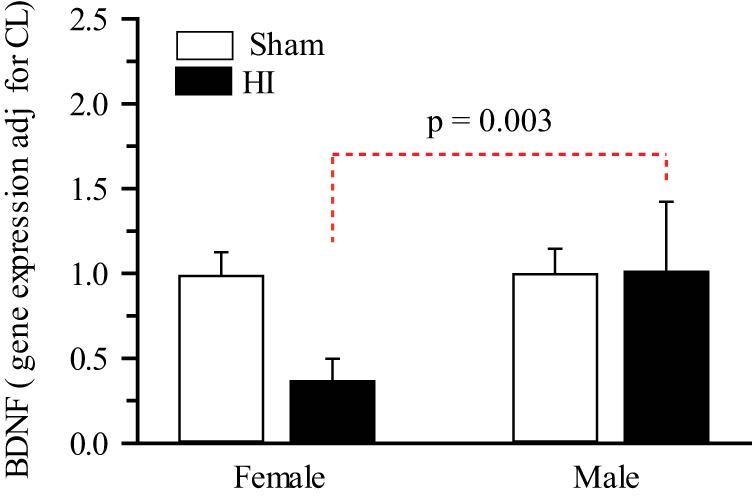
**BDNF gene expression are sexually differentiated in P9 neonatal hippocampi 3 d post-HI.** BDNF mRNA expression was quantified with qPCR in the hippocampi of male and female mice 3 d after sham operation or HI in ERα^+/+^ mice. Data is mean IL expression relative to sham female, adjusted for CL ± SEM, *n* = 5–6. *n*= number of pups.

Given that ERα mRNA and protein expression are increased in the female hippocampus post-HI and that genetic ablation of ERα abolishes HI-induced TrkB phosphorylation in both males and females, we investigated the possible mechanisms linking ERα and TrkB. One possibility would be through the classical ERα signaling pathway. This involves E_2_ binding to nuclear ERα resulting in DNA binding at estrogen response elements in the BDNF promoter region resulting in differentially increased BDNF expression in females. However, in our experiments BDNF mRNA expression was not increased, but instead significantly decreased in female hippocampus at 3 d post-HI (*p* =0.003^j^; Fig. 6). Given that the classical ERα pathway may not account for the crosstalk between the hippocampal ERα and TrkB, we then investigated non-classical ERα signaling which can involve rapid non-genomic signaling via membrane localized extranuclear ERα that is linked to rapid activation of signal-regulated kinases.

### Sexually differentiated expression of p-src^Y418^ in neonatal hippocampus is ERα dependent

Recent evidence suggests that there is a complex interplay of ERα with nerve growth factor receptor signaling that in part involves activation of the cellular tyrosine kinases called SFKs (Arpino et al., 2008). In this study, we proposed that activation of src, represents one of the initial steps in ERα-mediated cell signaling and that activation of SFK regulates TrkB in part by direct phosphorylation of TrkB at Y705/Y706 (Barletta et al., 2004; Huang and McNamara, 2010). Therefore, we determined the p-src^Y418^ expression in neonatal hippocampus 3 d post-HI as a surrogate marker for the SFK activity.

**Figure 7.**
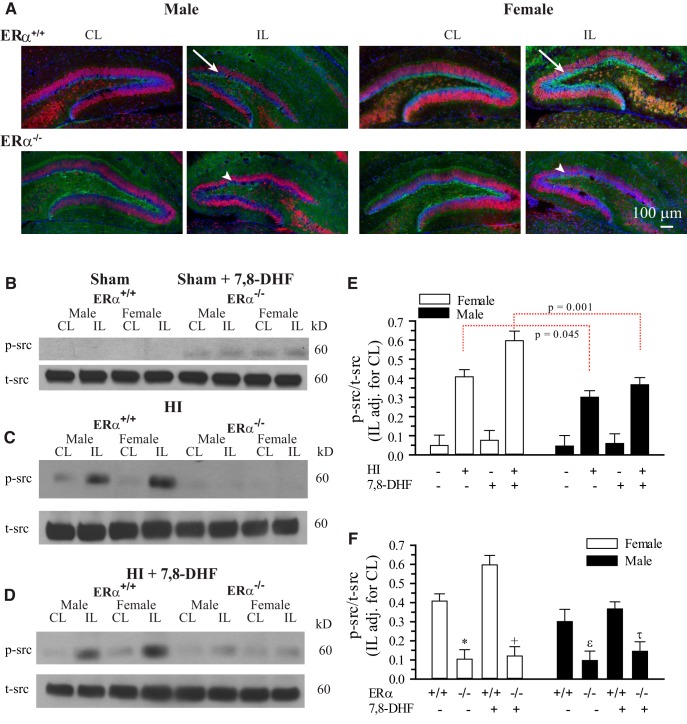
**Hippocampal p-src^Y418^ is sexually differentiated and ERα-dependent post-HI. *A***, Changes in the NeuN (red) and p-src^Y418^ (green) immunoexpression in the CL and IL dentate gyrus of ERα^+/+^ and ERα^−/−^ P9 mice examined at 3 d post-HI. Left two columns = male; right two columns = female; top row = ERα^+/+^; bottom row = ERα^−/−^. Arrow, Increased p-src^Y418^ staining. Arrowhead, Unchanged or decrease p-src^Y418^ in IL hippocampi. Scale bar, 200 μm ***B***, Representative blots of p-src^Y418^ and t-src from ERα^+/+^ male and female neonatal hippocampi. Mice were subjected to sham operation with and without 7,8-DHF therapy and the brains harvested 3 d later. ***C***, Representative blots of p-src^Y418^ and t-src from ERα^+/+^ and ERα^−/−^ male and female neonatal hippocampi. Mice were subjected to HI and the brains harvested 3 d later. ***D***, Representative blots of p-src^Y418^ and t-src from ERα^+/+^ and ERα^−/−^ male and female neonatal hippocampi. Mice were subjected to HI with 7,8-DHF therapy and the brains harvested 3 d later. ***E***, Summary figure of the IL p-src^Y418^/t-src ratio adjusted for CL in male and female hippocampi 3 d post-sham operation or post- HI with and without 7,8-DHF therapy. Data are mean IL adjusted for CL ± SEM, *n* = 3–5. *n* = number of blots per group (2 hippocampi per group). ***F***, Summary figure of the p-src^Y418^/t-src ratio in ERα^+/+^ and ERα^−/−^, male and female hippocampi 3 d post- HI with and without 7,8-DHF therapy. Data are mean IL adjusted for CL ± SEM, *n* = 3–5. *n* = number of blots per group. *p = 0.002^t^, +*p*= 0.001^u^ , ε*p*= 0.006^v^, τ *p* = 0.007^w^ versus ERα^+/+^.

Our results show a small increase in the p-src^Y418^ immunostaining in IL hippocampus of the male ERα^+/+^ mice 3 d post-HI (Fig. 7*A*, top row, left). By contrast, a robust increase in the p-src^Y418^ staining in the IL hippocampus of the female ERα^+/+^ mice occurred (Fig. 7*A*, top row, right). In the absence of ERα, this sex difference in HI-induced p-src^Y418^ expression was abolished (Fig. 7*A*, bottom row). To confirm these immunostaining findings, we detected p-src^Y418^ protein expression using immunoblotting (Fig. 7*B*–*D*). Immunoblotting results also showed that p-src^Y418^ protein expressions were significantly higher (1.5-fold) in female IL hippocampus post-HI (*p*=0.045^j^) and (1.6-fold) after 7,8-DHF therapy (*p* = 0.001^l^) compared with males post-HI (Fig. 7*E*). The sex differences seen in p-src^Y418^ with and without TrkB agonist therapy were abolished in ERα^−/−^ mice (*p* < 0.0001^m^; Fig. 7*F*) post-HI. We detected no statistically significant differences between the sexes in p-src^Y418^ expression in sham mice with or without 7,8-DHF therapy. These results reveal that phosphorylation of src^Y418^ is dependent on ERα and thus could mediate the ERα-TrkB crosstalk in neonatal mice hippocampus post-HI.

### Genetic ablation of ERα increases apoptosis in female hippocampus post-HI

**Figure 8.**
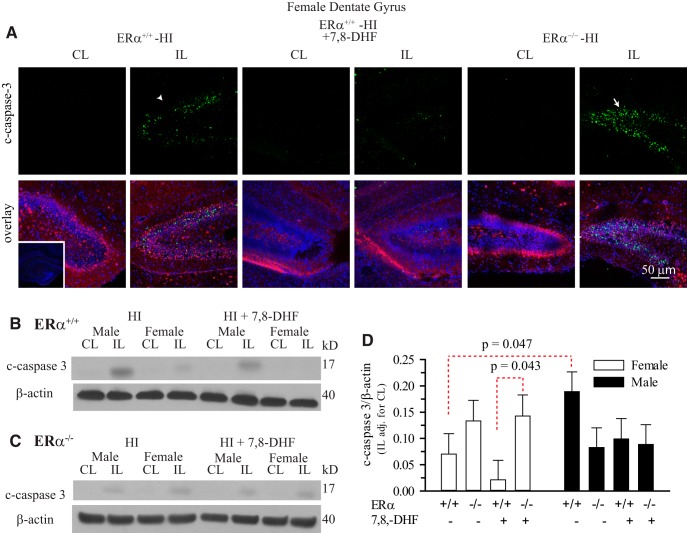
**TrkB agonist treatment decreases hippocampal apoptosis in ERα^+/+^ female mice but has no effect in ERα** ^−/−^ **female mice. *A***, Brain slices from female P9 mice were stained for c-caspase-3 (green), NeuN (red), and DAPI (blue) at 24 h post-HI. Left two columns = female ERα^+/+^; middle two columns = female ERα^+/+^ with 7,8-DHF therapy; left two columns = female ERα^−/−^. Arrow head, Increased c-caspase-3 staining in ERα^+/+^ IL dentate gyrus. Arrow, Augmented levels of c-caspase-3 staining in ERα^−/−^ IL dentate gyrus. Inset, Antibody control. Scale bar, 50 μm. ***B***, ***C***, Representative blots of c-caspase-3 in male and female neonatal hippocampi from ERα^+/+^ and ERα^−/−^with and without 7,8-DHF therapy 24 h post-HI. Blots were probed for β-actin as a loading control. ***D***, Summary figure of the IL c-caspase-3/β-actin ratio corrected for CL in ERα^+/+^ and ERα^−/−^ male and female hippocampi with and without 7,8-DHF therapy. Data are mean IL corrected for CL ± SEM, *n* = 3, *n* = number of blots per group (2 hippocampi per group).

TrkB signaling leads to activation of prosurvival pathways including PI3K/AKT, PLCγ-Ca^2+^, and Ras/MAPK (Minichiello, 2009). Thus, the enhanced responsiveness to the TrkB agonist therapy that we observe in female hippocampus would be expected to reduce post-HI apoptosis in these mice. Conversely, c-caspase-3 expression in mice in which ERα has been genetically ablated should be increased post-HI. To confirm this hypothesis, we investigated c-caspase-3 expression in the hippocampus of ERα^+/+^ and ERα^−/−^ mice with and without 7,8-DHF therapy at 1 d post-HI. As seen in Figure 8*A*, left, c-caspase-3 immunostaining was increased at 1 d post-HI in the female ERα^+/+^ mouse hippocampus. Interestingly, genetic ablation of ERα resulted in a marked increase in c-caspase-3 immunostaining in the IL female hippocampus (Fig. 8*A*, right). This suggests that ERα inhibits apoptosis in female neonatal hippocampus and contributes to neuroprotection post- HI. To further confirm these results, we quantified the c-caspase-3 protein expression in male and female hippocampus from ERα^+/+^ and ERα^−/−^ mice (Fig. 8*B*–*D*) at 1 d post-HI. We detected significantly higher c-caspase-3 expression in the hippocampus of ERα^+/+^ male mice compared with ERα^+/+^ female mice (*p* = 0.047^n^). In addition, TrkB agonist therapy mediated attenuation of c-caspase-3 levels observed in female ERα^+/+^ mice is abolished in the absence of ERα (*p* = 0.043^°^). Together, our data suggest that the sexually differentiated response to HI and HI + p-TrkB agonist therapy in the female neonate hippocampus is mediated by ERα and provides neuroprotection via facilitation of prosurvival cell signaling pathways downstream of TrkB phosphorylation. However, there were no differences detected in attenuation or exacerbation of the apoptosis when assessed by c-caspase-3 levels in male neonate hippocampus regardless of the TrkB agonist therapy or ERα status 1 d post-HI.

## Discussion

**Figure 9.**
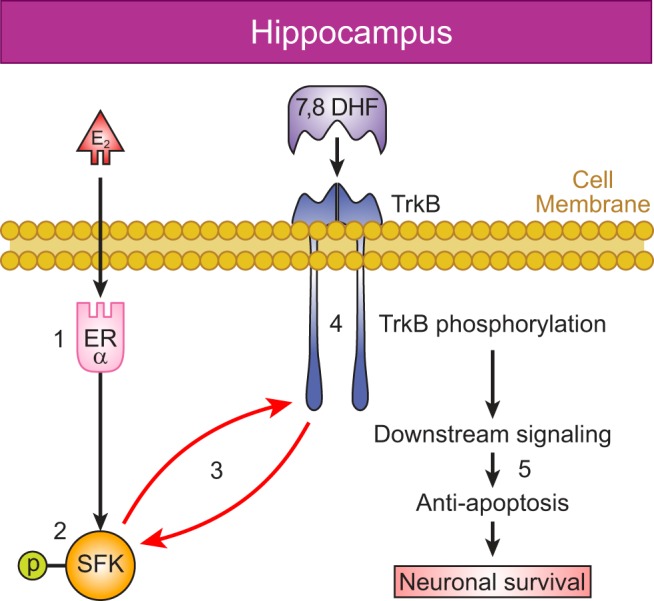
**Crosstalk between the ERα and TrkB post-HI. (*1*)** HI induces ERα expression in neonatal hippocampal cells, which are thereafter activated by circulating or neural-derived estradiol (E_2_). **(*2*)** ERα signaling prompts activation of SFK, and (***3***) SFK enhance phosphorylation and hence, enhance activation of TrkB receptors in response to (***4***) TrkB receptor ligands resulting in (***5***) decreased apoptosis and increased neuronal survival.

In this study, we tested the hypothesis that HI preferentially induces ERα expression in female neonatal hippocampus and that ERα is coupled to SFK activation that in turn augments phosphorylation of the TrkB and thereby results in decreased apoptosis and neuronal injury. Our studies confirm the validity of four key elements of this hypothesis, namely that: (1) HI differentially induces ERα expression in the hippocampus of the female versus male neonate, (2) src and TrkB phosphorylation post-HI is greater in females than in males after 7,8-DHF therapy, (3) src and TrkB phosphorylation post-HI depend upon the presence of ERα, (4) TrkB agonist therapy decreases the c-caspase-3 only in ERα^+/+^ female mice hippocampus (Fig. 9). These observations provide evidence that female-specific induction of ERα expression post-HI confers enhanced SFK activation, and thus enhanced neurotrophin signaling and neuroprotection with TrkB agonist therapy that may account for improved functional outcomes in female neonates following HI.

### Neuroprotective role of ERα post-HI

Using ERα and ERβ KO mice Dubal et al. (2001) demonstrated that the neuroprotective effects of E_2_ in adult rodents were dependent on ERα but not on ERβ after MCAO. It has also been reported that ERα is differentially increased in adult female cortex compared with males at the ischemia site after MCAO (Westberry et al., 2010). Although it has been shown that ERα is equally expressed in both naïve male and female neonatal rat hippocampus (Miranda et al., 1994;Solum and Handa, 2002; Pérez et al., 2003), the involvement of ERα in the neuroprotective effects of E_2_ in neonatal brains have not been studied. We report here that ERα expression is upregulated in the ischemic neonatal hippocampus in a sexually differentiated manner, with greater ERα mRNA and protein expression occurring in the female versus male neonate, in a manner similar to that previously reported in adult rodents at the ischemia site after MCAO (Westberry et al., 2010). The mechanisms that mediate this differential induction of ERα mRNA expression are unknown. One possibility is the epigenetic regulation of the ERα promoter region as it has been shown that the increase in expression of ERα mRNA in the adult female cerebral cortex following MCAO is due to demethylation of the ERα promoter in response to brain injury (Westberry et al., 2008). In adult males, there was no change in the methylation status of the ERα promoter which might explain the lack of increase in ERα mRNA in the cortex of male rats following MCAO.

### TrkB phosphorylation is more robust in the female neonate hippocampus post-HI

We have examined the possibility that the ERα expression is linked to decreased neurodegeneration and improved neurological outcome via interactions of ERα with TrkB signaling. Neurotrophins, such as BDNF, play critical roles in neuronal survival, neurogenesis, and synaptic plasticity (Waterhouse et al., 2012; Bothwell, 2014). Intracerebroventricular administration of BDNF produces rapid and robust increase in p-TrkB in the hippocampus and provides dramatic reduction in tissue loss 7 d post-HI in P7 rats. Similarly, BDNF pre-therapy in P7 rats results in improved spatial memory deficits and reduced caspase-3 activation at 30 d or 24 h post-HI, respectively (Almli et al., 2000; Han et al., 2000). However, the poor bioavailability of BDNF to the brain limits its therapeutic value (Poduslo and Curran, 1996) and small pharmacologic molecules were developed to activate TrkB exogenously such as 7,8-DHF (Jang et al., 2010), LM22A-4 (Han et al., 2012), adenosine A2A receptor agonists (Zeng et al., 2013), and TrkB-selective antibodies (Cazorla et al., 2011). Thus, pre-therapy with 7,8-DHF reduces stroke volume in adult male mice 2 d after MCAO (Jang et al., 2010) and reverses memory deficits in a mouse model of Alzheimer’s disease (Devi and Ohno, 2012). Similarly, the small molecule BNDF ligand LM22A improves functional outcomes in male mice after adult HI (Han et al., 2012) or TBI (Longo and Massa, 2013). The TrkB agonist antibody (27D7) pre-therapy enhances hippocampal neuronal survival and improves the long-term sensory motor function post-HI in neonatal rats (Kim et al., 2014). However, these studies did not investigate any role for sex differences in their neuroprotective outcomes. They were conducted either only in male animals or in pooled brains obtained from both males and females. In contrast, it has been reported that activation of TrkB with 7,8-DHF confers neuroprotection in female neonatal mice post-HI but not in males and this sex difference persists into adulthood as determined by hippocampal-dependent memory and learning at P60 (Uluc et al., 2013). In this study, we confirm the findings of Uluc et al. (2013) and report that the female neonate hippocampus has statistically significantly higher levels of TrkB phosphorylation with 7,8-DHF therapy post-HI. This effect was not found to be attributed to the TrkB subtypes such as to the t-TrkB, which can act as a dominant-negative inhibitor of f-TrkB and prevent f-TrkB from getting phosphorylated (Fenner, 2012). Together, our data suggest that HI and 7,8-DHF therapy increases hippocampal p-TrkB expression in a sexually differentiated manner and that the increased hippocampal p-TrkB expression is associated with increased hippocampal ERα expression.

### Hippocampal TrkB phosphorylation depends on the presence of the ERα

Estradiol therapy has been shown to increase the hippocampal TrkB phosphorylation in adult mice, but this response is ablated in ERα^−/−^ mice (Spencer-Segal et al., 2012). We therefore tested the hypothesis that the sexually differentiated increase in hippocampal TrkB phosphorylation in the neonate following HI is a consequence of increased ERα signaling (Fig. 9). We found that HI and 7,8-DHF therapy failed to increase the hippocampal p-TrkB levels in ERα^−/−^ mice 3 d post-HI. These results clearly demonstrate that the hippocampal TrkB phosphorylation depends upon the presence of ERα. When coupled with the observation that ERα expression is greater in the female hippocampus post-HI, these findings provide strong evidence that the sexually differentiated pattern of phosphorylation of TrkB following HI and after TrkB agonist therapy is mediated by ERα. The hippocampal E_2_ action could be exerted through increased production of a TrkB ligand or through stimulation of intracellular signaling pathways following HI in neonatal brains (McDevitt et al., 2008; Levin, 2014). To test the first possibility, we measured the hippocampal BDNF mRNA levels as an indirect indication of the nuclear ERα signaling in neonatal mice following HI. Our findings showed that hippocampal BDNF mRNA levels decreased in the female hippocampus compared with male hippocampus. This result suggests that the first possibility of E_2_ inducing increased production of intrinsic TrkB ligand via classical pathway cannot be held responsible for increased TrkB phosphorylation. Thus, we hypothesized that the crosstalk between the ERα and TrkB is through the cytoplasmic kinase signaling after rapid membrane ERα activation.

### Crosstalk between ERα and TrkB might be mediated by p-src in neonatal hippocampus post-HI

One possible mechanism that can link the ERα with TrkB is that ERα can couple to the cytoplasmic SFKs through the membrane initiated signaling resulting in phosphorylation of the TrkB (Levin, 2012). Using mixed neuronal/glial cortical cultures, Huang et al. demonstrated that the SFKs can directly phosphorylate TrkB^Y705/Y706^. In addition, the fact that TrkB immunoprecipitates with both src and fyn confirms a physical relationship between these kinases and TrkB (Huang and McNamara, 2010). Our results are consistent with a role for membrane-initiated rapid signaling of ERα. The expression of p-src significantly increases in female hippocampus compared with males following HI and 7,8-DHF therapy. This sex difference seen in p-src expression in the hippocampus is totally ablated in ERα^−/−^ mice. Thus, increases in ERα expression in female hippocampus could lead to increases in signaling through p-src, which could facilitate phosphorylation of TrkB and promote downstream anti-apoptotic signaling post-HI (Fig. 9).

### TrkB agonist therapy decreases apoptosis in female hippocampus post-HI

The mechanisms underlying the sex differences in neonatal brains post-HI is unknown. Recent experiments into sex-dependent cell death pathways in neonates have led to a hypothesis that in females cell death occurs primarily through a caspase-dependent pathway, whereas in males it is secondary to PARP activation post-HI (Lang and McCullough, 2008). Genetic deletion of PARP protected males but not females post-HI in P7 mice (Hagberg et al., 2004). Thus, Renolleau et al. (2007) reported that c-caspase-3 in females is elevated 1 d post-HI in the cortex of P7 rats and that the pan-caspase inhibitor Q-VD-OPh is neuroprotective in females, but not in males. In our study, unlike the results of Renolleau et al. (2007), we detected higher c-caspase-3 protein expression in male hippocampus compared with females at 1 d post-HI. The reason for this apparent discrepancy could be the result of sex differences in regional caspase expression postinjury (hippocampus vs cortex), although further studies are required to confirm this finding. Consistent with our hypothesis, TrkB agonist therapy resulted in a significant decrease in c-caspase-3 in female hippocampus, but not in males. In addition, c-caspase-3 expression increased in the female ERα^−/−^ mice hippocampus and 7,8-DHF therapy did not have any effects decreasing the apoptosis. In contrast, neither 7,8-DHF therapy nor ERα deletion changed c-caspase-3 expression in the male hippocampus. This could be the result of different apoptotic pathways present in the male hippocampus that may not be caspase dependent. Future studies are required to elucidate that relationship between sex-dependent cell death pathways and ERα-dependent neurotrophin signaling.

## Conclusion

To our knowledge, this is the first study to reveal that hippocampal ERα signaling is integrated with TrkB signaling in a manner that it is more robust in neonatal females, potentially accounting for the sex differences seen after HI-related brain injury. We have shown that genetic deletion of ERα abolishes the sexually differentiated expression of p-TrkB in response to its agonist, 7-8, DHF, in neonatal mice post-HI. In addition, we demonstrated that there is a sex difference in the post-HI activation of p-src in neonatal hippocampus that is also ERα dependent. Thus, increased ERα signaling through src in female hippocampus that facilitates TrkB signaling resulting in decreased c-caspase-3 is a novel mechanism that could account for the sex differences in brain injury following HI in neonates. Better understanding of the mechanisms involved in female resistance to injury could lead to targeted therapies in neonates following hypoxic and ischemic encephalopathy.

## Synthesis

The decision was a result of the Reviewing Editor Jun Chen and the peer reviewers coming together and discussing their recommendations until a consensus was reached. A fact-based synthesis statement explaining their decision and outlining what is needed to prepare a revision is listed below. The following reviewer(s) agreed to reveal their identity: Margaret McCarthy

This manuscript examined the mechanism of a somewhat controversial notion of gender difference in vulnerability to perinatal hypoxic-ischemic (HI) injury. The authors proposed that the gender difference in estrogen receptor ERα (higher expression on females) modulates TrkB-mediated protective mechanisms. The authors use a modified version of the Vannuci model in PN9 mice. The study appears to be well executed and the manuscript is well presented. The work is important both for its illustration of sex differences in response to injury and exploration of the mechanism by which that occurs, as well as the identification of potential therapeutic targets that might be utilized in a sex specific manner, or adapted to benefit both sexes.

Reviewer 1 has a few relatively minor concerns about the manuscript, which should be handled by the authors easily:

1. The Discussion is longer than it need be and reiterates much that has already been said, I recommend a robust streamlining.

2. Throughout the Results the authors refer to a "sexually differentiated" response or effect. But sexual differentiated refers to a specific developmental process and proving it has occurred requires specific experiments involving either hormone replacement or control for sex chromosome complement. It would be much more accurate to change such references to "sex differences".

3. Figure 5C - it is surprising there is not a significant difference in f-TrkB, certainly there must be a strong trend?

4. The photomicrographs in Figure 7A are confusing, it's hard to know what the arrow heads are pointing at, the images look to be from different portions of the hippocampus, just overall not very convincing.

5. Introduction line 33 - move "resulting" to behind the references.

Reviewer #2 is more critical, having several major-moderate concerns that need to be addressed by the authors. Accordingly, a few more appear-to-be straightforward experiments may be necessary to fully support their conclusions:

1. (MAJOR) What is the ligand for ERα? Estrogen? Can the authors show gender difference in estrogen level between P9 male and female mice?

2. (MAJOR) Most of the analysis is focused on DG, but as far as this reviewer knows, DG is the most frequently damaged area in the Vannucci HI model. Why do the authors focus their analysis in this region? Do they see similar gender difference in other parts of the hippocampus and the cortex? Please provide such results.

3. (Moderate) Figure 8A showed nice reduction of active Caspase-3 by TrkB agonist and induction in ERα-knockout mice in female. What happen to male pups under the same treatment? Please provide.

4. (Minor) Is there difference in TrkB expression between male and female mouse pups, either with or without HI insult.
